# Public health significance of the white-tailed deer (*Odocoileus virginianus*) and its role in the eco-epidemiology of tick- and mosquito-borne diseases in North America

**DOI:** 10.1186/s13071-025-06674-6

**Published:** 2025-02-06

**Authors:** Ilia Rochlin, Joan Kenney, Eliza Little, Goudarz Molaei

**Affiliations:** 1 Stony Brook University, Stony Brook, NY USA; 2 Centers for Disease Control and Prevention, Fort Collins, CO USA; 3 Connecticut Department of Public Health, Hartford, CT USA; 4 Connecticut Agricultural Experiment Station, New Haven, CT USA; 5 Yale Uinversity, New Haven, CT USA

## Abstract

White-tailed deer (*Odocoileus virginianus*) are a ubiquitous species in North America. Their high reproductive potential leads to rapid population growth, and they exhibit a wide range of biological adaptations that influence their interactions with vectors and pathogens. This review aims to characterize the intricate interplay between white-tailed deer and the transmission cycles of various tick- and mosquito-borne pathogens across their range in the eastern United States and southeastern Canada. The first part offers insights into the biological characteristics of white-tailed deer, their population dynamics, and the consequential impacts on both the environment and public health. This contextual backdrop sets the stage for the two subsequent sections, which delve into specific examples of pathogen transmission involving white-tailed deer categorized by tick and mosquito vectors into tick-borne and mosquito-borne diseases. This classification is essential, as ticks and mosquitoes serve as pivotal elements in the eco-epidemiology of vector-borne diseases, intricately linking hosts, the environment, and pathogens. Through elucidating these associations, this paper highlights the crucial role of white-tailed deer in the transmission dynamics of tick- and mosquito-borne diseases. Understanding the interactions between white-tailed deer, vectors, and pathogens is essential for effective disease management and public health interventions.

**Graphical Abstract:**

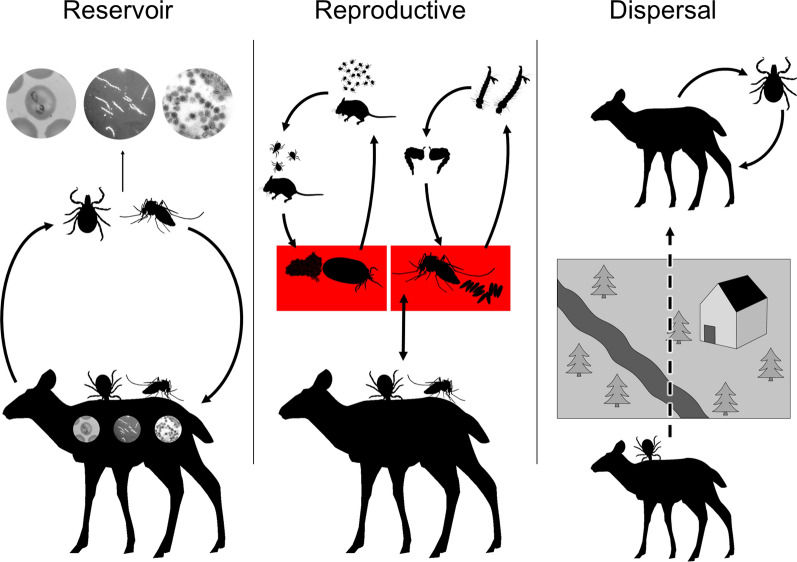

## Background

White-tailed deer (*Odocoileus virginianus*, Zimmermann, hereafter WTD) are a ubiquitous species, with as many as 38 different subspecies found from central Canada, over most of the US, and in northern South America (Colombia and Venezuela) [1]. This review focuses on eastern North America, because, in Central and South America, the relationship between WTD and vector-borne pathogens remains poorly understood [2]. In eastern North America, adult WTD typically measure around 130–230 cm (4.3–7.5 ft) in length from nose to tail tip and weigh 40–150 kg (88–330 lbs) [3]. White-tailed deer exhibit a wide range of biological adaptations that influence their interactions with ticks, mosquitoes, and the pathogens they transmit [4]. These ungulate mammals are herbivore generalists, preferring edge habitats (ecotones) between forests, agricultural or grasslands, and even suburban/exurban landscapes, providing ample forage [3]. White-tailed deer can live up to 18 years, and females, or does, often produce two fawns each year, with the potential to produce 30 offspring in their lifespan [3, 5]. The high reproductive potential of WTD leads to rapid population growth, with populations doubling in size every 2–3 years [5, 6]. Home range sizes for bucks (male WTD) can range from about 162 to 810 hectares (approximately 400 to 2000 acres), although in some cases they may have even larger ranges, especially during the breeding season, while those for does are generally smaller, typically ranging from about 40 to324 hectares (approximately 100 to800 acres) [7]. However, the home range can vary depending on factors such as habitat quality and population density. White-tailed deer can disperse over significant distances. Studies have documented dispersal movements of WTD ranging from a few kilometers to > 50 km [8]. These dispersal patterns are influenced by factors such as habitat quality, population density, and landscape connectivity.

Ecological studies have revealed the complex dynamics of WTD populations, influenced by habitat fragmentation, predator–prey relationships, and human activities [9]. High WTD densities in fragmented landscapes can increase contact rates among hosts and vectors, thereby facilitating disease transmission. Moreover, WTD browsing behavior can alter vegetation composition and create favorable conditions for tick habitat, impacting disease risk [9–11]. The biological carrying capacity of WTD is defined as the number of individuals that a given area can support over an extended period and is determined by food resources and habitat requirements [12, 13]. White-tailed deer populations become ecologically problematic when densities exceed 8 WTD/km^2^ (approximately 21 per square mile), leading to habitat quality decline and herd health issues [12, 14, 15].

## White-tailed deer

### Historical context of white-tailed deer in the eastern US

In the eastern US, beginning in the late seventeenth century, forests were felled for timber, farming, and charcoal production [16, 17]. Deforestation coupled with unregulated, year-round hunting of WTD throughout the eighteenth and nineteenth centuries resulted in the near extirpation of WTD [18–21]. Remnant populations survived in mountainous areas, coastal marshes, and other places inaccessible to loggers and hunters. The abandonment of farmland and movement of people westward in the late nineteenth century led to the progression of open areas into early successional forests—ideal habitat for WTD. With reforestation, hunting restrictions, predator suppression, and WTD reintroduction from remnant populations, WTD populations started to rebound in the mid-1900s [19, 20, 22, 23].

Fewer predators, both wild and human, have resulted in continued WTD population growth and changed their behaviors, such as foraging and bedding on residential properties [9, 24, 25]. Since reforestation over the last century, human encroachment into forested areas (suburbia and exurbia) has led to fragmented woodlands [10, 26]. Like early successional forests, fragmented forests with edge habitat between forested and open residential and agricultural areas support high densities of WTD [27]. Present-day forests are more mature and have reduced structural complexity, being generally the same age [28]. Thus, WTD largely depends on residential and agricultural landscapes for foraging to meet their energy requirements [29].

As suburbia and exurbia continue to expand, managing WTD populations becomes more challenging. White-tailed deer are well adapted to suburban environments, and residential areas remain largely inaccessible to hunters, resulting in a high potential for WTD-human conflict [30]. The greening of urban areas, through green spaces and habitat corridors, has led to transient and established populations of WTD and (re)introduction of vectors and vector-borne diseases in urban parks due to closer contacts between humans and WTD [31, 32].

### White-tailed deer impacts on ecosystem and wildlife health

White-tailed deer, at high densities, are true ecosystem engineers, contributing to a cascade of ecological consequences through over-browsing, including the promotion of non-native plants, the depletion of native wildflowers and other native understory plants, and the prevention of forest regeneration [9, 15, 33–36]. By shaping the diversity and structure of plant communities, WTD impacts other animals in the ecosystem, resulting in changes to small mammal and bird communities as well as insect and disease outbreaks [3, 12, 15]. Some reports have shown that, even long after WTD reduction efforts, historically, overabundant WTD have lasting effects on plant and animal communities [9, 11, 15, 33, 35, 37]. The health of forests depends on maintaining and/or promoting species diversity and resilience, especially when considering the additional threats of invasive species and a changing climate. Therefore, it is essential to the health of forests that WTD densities do not exceed their biological carrying capacity [9, 11, 12, 14, 15].

### White-tailed deer-human interactions

The US WTD population reached its nadir in 1900 with 350,000 WTD but has since grown to upwards of 34 million [38]. Present-day WTD densities are much higher than in the past [3, 19, 20, 36, 39]. White-tailed deer harvests by hunters have been stable since the 1990s but may be decreasing in some areas. Nationwide, there were 11.5 million hunters in 2016, compared to 13.7 million in 2011, and recruitment and retention of new and younger hunters have also decreased in recent years [40]. High WTD density exacts economic costs to crops and ornamental gardens and through vehicle collisions. In the US, WTD-vehicle accidents cause about 29,000 human injuries, 150 human fatalities, and 1 billion dollars in property damage annually [41].

Human tolerance for WTD and cultural carrying capacity can outweigh biological carrying capacity, making it difficult for communities to reach consensus about reducing WTD herds to low densities [42]. Because the ecological damage done by WTD is slow-moving and cumulative, it is often discounted, or worse, believed to be a natural process [33].

Successful WTD management requires consideration of the ‘human dimensions’ of management, defined as the public’s diverse views and values about these animals and their impacts [23]. White-tailed deer are valued for watching, hunting, and contributing to the economy. In Maine, USA, support for WTD reduction was motivated by the negative consequences of the overabundance of these animals, including vehicle collisions, agriculture and garden damage, and forest health, as well as contributing to vector-borne diseases [43]. Management programs, therefore, should be developed to both educate the public and define WTD management goals to maintain their populations at biological carrying capacity. White-tailed deer management will not be solved with a single silver bullet approach; rather, solutions need to account for various stakeholder concerns and be multi-pronged to succeed [44, 45].

### White-tailed deer's impact on public health issues

Vector abundance is linked to the abundance of their vertebrate hosts [10, 20, 46]. White-tailed deer are key hosts to vectors that impact humans, domestic animals, and wildlife [20]. White-tailed deer populations are associated with the spread and density of common tick vectors, including *Ixodes scapularis*, *Amblyomma americanum*, *Amblyomma maculatum*, *Rhipicephalus annulatus*, and *Haemaphysalis longicornis* [20, 47, 48]. White-tailed deer have also been identified as the single most important source of blood for mosquito species, including *Anopheles quadrimaculatus*, *Anopheles punctipennis*, and *Aedes canadensis*, or the most frequent mammalian host for several other mosquito species, such as members of the *Culex pipiens* complex, *Culex salinarius*, *Coquillettidia perturbans*, and *Aedes vexans* [49–52]. These mosquito species have also been identified as principal, secondary, or bridge vectors of some of the most important arboviruses in the northeast US. The role of WTD in the ecology and transmission dynamics of mosquito-borne arboviruses, including West Nile virus (WNV) and eastern equine encephalitis virus (EEEV), is not clearly understood, but it has been postulated that widespread abundance and distribution of these ungulate mammals in some localities could be zooprophylactic by diverting mosquito feeding from avian amplifying hosts [50].

**Fig. 1:**
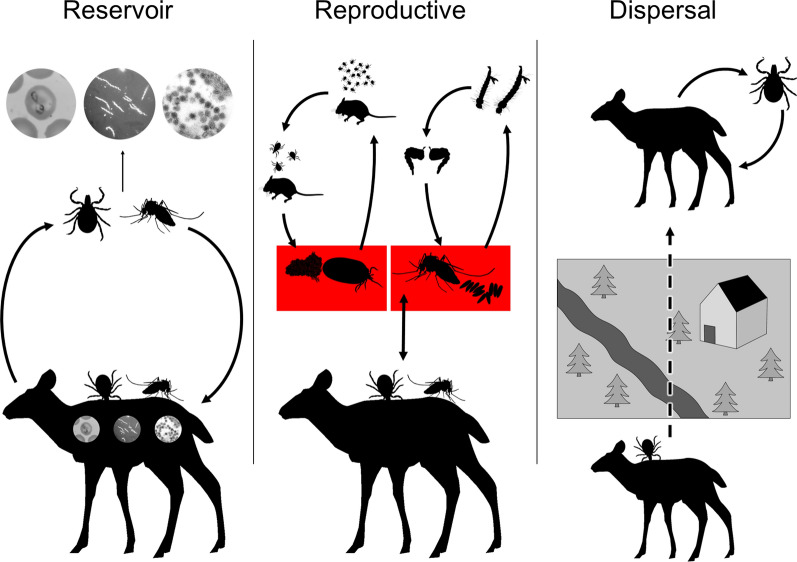
Roles of white-tailed deer in tick- and mosquito-borne pathogen transmission. A reservoir host maintains the infectious vector-borne agents (such as bacteria, viruses, or parasites) within their populations or supports direct passing of pathogens between vectors during cofeeding, thus perpetuating the cycle of transmission. A reproductive host provides a suitable environment for vectors to complete their reproductive cycle, serving as the main source of blood meals for adult vectors and allowing them to mate and lay eggs. Unlike reservoir hosts, a reproductive host does not support direct transmission of pathogens; however, it plays a crucial role in the life cycle of vectors, facilitating vector population growth. A dispersal host for ticks is an animal species that aids in the movement or dispersal of tick populations from one location to another. Unlike mosquitoes, ticks cannot travel long distances on their own, so they rely on hosts to transport them to new habitats or geographic regions

**Fig. 2:**
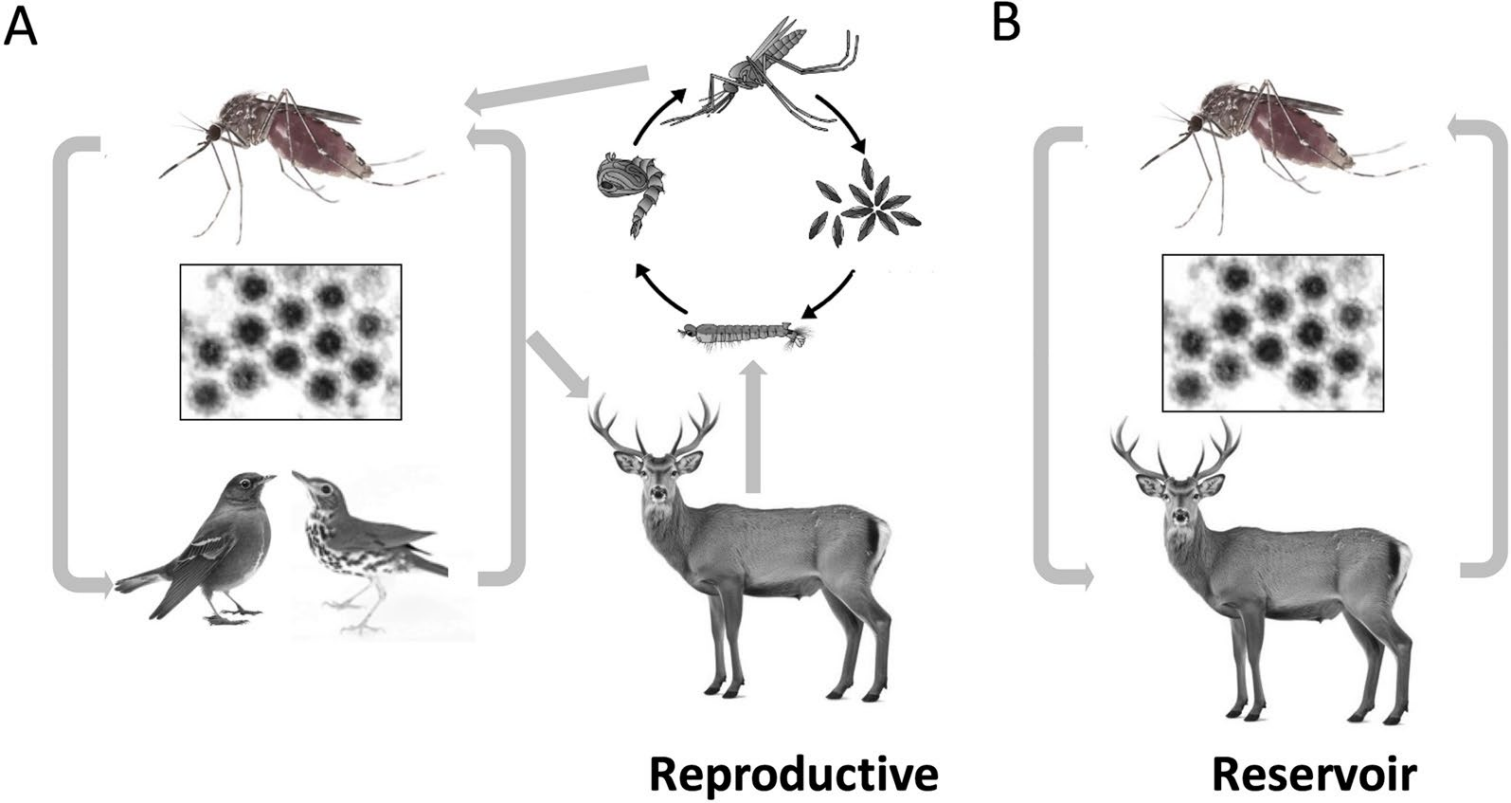
How mosquitoes utilize white-tailed deer as reproductive and reservoir hosts. **A** A representative example of virus maintenance in an alternating primary vector-host (bird in this case) cycle with a secondary vector utilizing deer as a reproductive host. **B** A depiction of a cycle in which the WTD serves as the viral reservoir host and vector blood meal source in the vector-host cycle

In this article, we describe the multiple roles WTD play as reservoir, reproductive, and dispersal hosts of tick and mosquito vector populations and associated vector-borne diseases (Fig. 1). In the context of tick- and mosquito-borne pathogens, a reservoir host refers to an animal species that supports a habitat for vector survival and reproduction, serving as a source of blood meals (Fig. 2). A key ecological role of reservoir hosts is to maintain the infectious vector-borne agents (such as bacteria, viruses, or parasites) within their populations or to support direct passing of pathogens between vectors during cofeeding, thus perpetuating the cycle of transmission. A reproductive host provides a suitable environment for vectors to complete their reproductive cycle, serving as the main source of blood meals for adult vectors and allowing them to mate and lay eggs. Unlike reservoir hosts, a reproductive host does not support direct transmission of pathogens; however, it plays a crucial role in the life cycle of vectors, facilitating their reproduction and population growth. A dispersal host for ticks is an animal species that aids in the movement or dispersal of tick populations from one location to another. Unlike mosquitoes, ticks cannot travel long distances on their own, so they rely on hosts to transport them to new areas. A dispersal host transports ticks over significant distances, potentially spreading to new habitats or geographic regions. This movement contributes to the dispersion and colonization of tick populations as well as the transmission of tick-borne diseases to new areas. In this article, we illustrate the three roles of WTD in pathogen transmission by providing examples of representative pathogens responsible for the most prevalent human tick- or mosquito-borne diseases.

Eradication of a zoonotic vector-borne disease is not possible without reducing the reproductive or pathogen reservoir host. One of the key insights gleaned from research is the existence of a WTD density threshold for tick-borne disease transmission. When WTD populations exceed certain thresholds, estimated at approximately 5–7 animals per km^2^, the risk of tick-borne diseases escalates significantly [46, 53]. This threshold serves as a crucial metric for wildlife managers and public health officials to implement effective strategies for disease control. By managing WTD populations within or below these thresholds, communities can mitigate the risk of tick-borne diseases and safeguard public health [54]. Reduction of WTD densities could simultaneously address concerns over ecosystems, wildlife, and human health [9, 11, 12, 20, 23, 33, 36, 46, 53–55].

### Conclusions: white-tailed deer

White-tailed deer play a pivotal role in shaping ecosystems, influencing wildlife health, and driving public health concerns. Their high reproductive capacity and adaptability to various environments, including suburban and agricultural landscapes, have led to rapid population growth and significant ecological impacts. Overabundant WTD populations not only disrupt plant communities and forest regeneration but also facilitate the proliferation of vectors, such as ticks and mosquitoes, which transmit various zoonotic diseases. We describe the multiple roles WTD play as reservoir, reproductive, and dispersal hosts of tick and mosquito vector populations and associated vector-borne pathogens (Fig. 1), highlighting the integral role of WTD in vector ecology. Managing WTD populations is crucial for mitigating these cascading effects on ecosystems, wildlife, and human health. The density thresholds for disease transmission, particularly in the case of tick-borne illnesses, underscore the need for effective population control strategies. Keeping WTD densities below critical thresholds not only reduces the risk of vector-borne diseases but also helps restore diversity and resilience in forested ecosystems. Comprehensive management approaches—integrating public education, wildlife management, and public health initiatives—are essential for maintaining sustainable WTD populations and mitigating their negative impacts.

## White-tailed deer's relationship with ticks and tick-borne pathogens

### White-tailed deer as a reservoir host

#### Representative tick-borne human disease: Ehrlichiosis

##### Public health impacts

Human monocytic ehrlichiosis (HME) is caused by *Ehrlichia chaffeensis* and *E. ewingii* that are transmitted by lone star ticks (*Amblyomma americanum*) [56–58]. From 2001 to 2019, the number of reported cases of HME increased nearly 15-fold from 142 in 2001 to 2093 in 2019, and then a substantial decrease in the number of disease cases was observed in 2020 (*n* = 1178), likely due to the COVID-19 pandemic, and the cases remained lower than in the pre-pandemic level in 2021 (*n* = 1337) [56]. However, there is convincing evidence that the number of human disease cases is grossly underreported [57, 59, 60]. In areas endemic for ehrlichiosis and with high populations of lone star ticks, it is likely that the vast majority of the human cases (up to 99% by some estimates) are not recognized and diagnosed [60]. The reasons for such underreporting or misdiagnosis included lack of awareness about ehrlichial disease and non-specific influenza-like symptoms in mild cases [57, 60]. In more serious cases, patients with HME can develop central nervous system impairment or septic shock-like syndrome [57]. HME can be severe in immunocompromised individuals, with a fatality rate reaching 3% [57]. In high-risk areas, the incidence rate can be as high as 4.7/100,000 [59].

##### Pathogen

*Ehrlichia chaffeensis* was described in 1991 as a novel, previously unrecognized pathogen [61]. Rickettsiales family Anaplasmataceae, a family of microorganisms closely related to rickettsias, includes other intracellular pathogens from the genera *Ehrlichia* and *Anaplasma* [57, 58]. *Ehrlichia ewingii* is another *Ehrlichia* pathogen that can cause human disease and is transmitted in a cycle similar to that of *E. chaffeensis* [20, 62].

##### Vector

Lone star tick (*A. americanum*) is a three-host hard tick distributed from west-central Texas north to Iowa and eastward in a broad belt spanning the southeastern US into the coastal areas as far north as New England [63]. Lone star ticks are notorious for their aggressive feeding behavior, with all three stages biting humans [58]. The lone star tick is a competent vector capable of transmitting *Ehrlichia* pathogens to WTD [64].

##### Role of white-tailed deer

Although lone star ticks feed on a variety of medium- and large-sized mammals, WTD are the principal or “keystone” hosts for all stages of this tick species [20, 65, 66]. At the peak season of *A. americanum* density, approximately 200 adult ticks, 500 nymphs, and 1500 larvae were carried by each WTD on average [67]. Arguably, the strongest evidence on the importance of WTD as a key host for maintaining high *A. americanum* abundance is provided by exclusion and control studies. White-tailed deer's exclusion from patches of habitat resulted in significant reductions of questing lone star tick populations ranging from approximately 90% in the larval abundance to 50% for nymphs and adults [68, 69]. Treating WTD with acaricide using four-poster devices generally led to reductions in questing *A. americanum* populations of > 90% for larvae and 50–90% for nymphs and adults, depending on the site and the year [70–72].

Linear dependency of *A. americanum* and WTD abundance was also notable in computer simulations [73], allowing the development of a predictive surveillance system for *E. chaffeensis* [74]. This model was based on the established concept of *E. chaffeensis's* natural cycle between WTD and lone star ticks. Isolations of *E. chaffeensis* from WTD blood provided strong initial support for this hypothesis [75]. Further support was furnished by the experimentally infected WTD with *E. chaffeensis* and observing the persistent presence of the bacteria in the blood for several weeks [76]. Finally, transmission of *E. chaffeensis* from lone star ticks to WTD was also experimentally demonstrated [64].

Based on numerous studies across the eastern US, *E. chaffeensis* prevalence varied from approximately 5–15% in lone star tick populations and 7–54% in WTD (reviewed in [20]). Lone star ticks were also capable of transmitting *E. ewingii* to WTD [64]. The prevalence of *E. ewingii* in vectors and hosts appeared to be lower than that of *E. chaffeensis*, ranging from approximately 1–10% in lone star tick populations and 4–20% in WTD (reviewed in [20]).

### Other tick-borne pathogens with similar transmission cycle

At present, *E. chaffeensis* is the only known pathogen for which WTD has been definitively identified as a reservoir host. Other pathogens, such as closely related *E. ewingii*, the causative agent of granulocytic ehrlichiosis, and *Borrelia miyamotoi*, a spirochete responsible for hard tick relapsing fever and transmitted by blacklegged ticks, may also be circulating in a similar transmission cycle between WTD and ticks [77, 78]. However, further research is necessary to elucidate the importance of WTD in the transmission cycles of these pathogens [23].

Another pathogen for which WTD may play a reservoir-like role is Powassan virus (POWV; Flaviviridae, Orthoflavivirus), or *Orthoflavivirus powassenense*, which is the only member of the tick-borne encephalitis virus serogroup in North America [79, 80]. Phylogenetically, POWV is represented by two lineages. Lineage I is estimated to have diverged from European tick-borne virus strains between 2000 and 6000 years ago [81], whereas lineage II, sometimes referred to as deer tick virus, was initially recognized and described in 1997 [82]. Historically, lineage I viruses have been associated with species of ticks less likely to associate with humans, such as *Ixodes cookei*, while lineage II viruses are primarily found in *I. scapularis*, which is a known vector of this pathogen for humans [82]. Neutralizing antibodies to POWV have been prevalent in WTD in New England, mid-Atlantic, and Midwestern states in the US [83, 84]. In Europe, tick-borne encephalitis virus is thought to be maintained primarily by transmission among cofeeding ticks on the same host [85]. White-tailed deer is the only host species able to support a large number of adult cofeeding female *I. scapularis*, which may be conducive to a similar mechanism of transmission [23, 86]. Recently, cofeeding transmission has been demonstrated for POWVs and two tick vector species, *I. scapularis* and *H. longicornis* (longhorned tick) [87]. While this study was conducted using a laboratory mouse model, adult ticks of both species feed primarily on WTD under natural conditions, suggesting a possibility of cofeeding transmission, which should be the focus of future studies.

### White-tailed deer as a reproductive host

#### Representative tick-borne human disease: Lyme disease

##### Public health impacts

Transmission of Lyme disease spirochete to humans by a tick bite was conclusively demonstrated in the early 1980s [88, 89]. Since its discovery, reported Lyme disease human cases in the US increased from about 226 in 1981 to over 63,000 in 2022 [90]. However, the actual public health burden of Lyme disease is estimated to be much higher. Lyme disease has become one of the most common infections in the US, with the number of cases tripling between 2004 and 2016 to 240,000–444,000 annually [91–93], with incidence rates reaching as high as 83/100,000 in “high-risk” states [94, 95]. Fourteen “high-risk” northeastern, mid-Atlantic, and upper Midwest states accounted for approximately 96%, or close to 200,000, confirmed Lyme disease cases in 2008–2015 [94]. Nearly half a million patients were diagnosed with and treated for Lyme disease in recent years [95, 96]. While it is difficult to assess the economic impacts on healthcare systems, the cost estimates for Lyme disease ranged from approximately $800 million to > 1 billion dollars annually [97, 98].

##### Pathogen

The spirochete responsible for Lyme disease was described in 1982 [88, 89] and assigned the name *Borrelia burgdorferi* in 1984 [99]. Lyme borreliosis is caused by various strains or genospecies of *Bo. burgdorferi* [100]. Recently, a new revision of the genus *Borrelia* based on genetic analysis proposed to split the genus into two genera: *Borrelia* and *Borreliella*, the latter containing the members of the Lyme disease *Bo. burgdorferi* [101]. Thus, *Borrelia* sensu lato includes spirochetes that cause relapsing fever and are primarily transmitted by soft-bodied ticks (Argasidae) and lice. In contrast, *Borreliella* species are associated with Lyme borreliosis and are transmitted primarily by hard-bodied ticks (Ixodidae). This revision, however, is controversial and has not been widely accepted [102]. As a result, the debate on the status of the genus *Borrelia* has not been settled [89].

##### Vector

The blacklegged tick (*Ixodes scapularis*) is the main vector of Lyme disease in the US [103]. The deer tick (*Ixodes dammini*) of the northeastern US was described as a separate species in the scientific literature predominantly between 1979 and 1993 [104, 105]. However, it was shown genetically to be conspecific with the blacklegged tick (*I. scapularis*), and the name is no longer in use [106]. *Ixodes scapularis* has a 2-year, three-host species life cycle: larvae feed on small mammals and reptiles, while nymphs attack a very broad range of mammalian and bird species [107–111]. Nymphal ticks are primarily responsible for Lyme disease transmission to humans and animals [107–111].

##### Transmission cycle

The main reservoir for *Bo. burgdorferi* in the northeastern US is the white-footed mouse, *Peromyscus leucopus* [10, 105, 107, 112, 113]. Over 90% of larval and nymphal blacklegged ticks fed on white-footed mice in an endemic Lyme area in New England [111], and nearly one-half of the larvae were infected with *Bo. burgdorferi* [113]. One of the most prominent aspects of the blacklegged tick ecology is the broad range of host species attacked by larvae and nymphs: at least 31 mammalian species, 49 bird species, and 4 lizard species have been recorded [107, 110]. The importance of host species, therefore, is determined by density and the number of infected ticks produced by individuals of a host species. The white-footed mouse’s high abundance and ability to infect a large proportion of larvae feeding on this species make it the principal reservoir for *Bo. burgdorferi* compared to other abundant rodent hosts such as voles and chipmunks [113, 114]. Other small mammals, such as shrews and chipmunks, can support the *Bo. burgdorferi* enzootic transmission cycle in areas with low populations of white-footed mice [115]. Medium-sized mammalian species, including rabbits, raccoons, and opossums, play only a minor role, if any, in the transmission cycle [10, 107, 115]. In the southern part of the *I. scapularis* range (south of Virginia), human Lyme disease is rare or absent due to the different ecology and behavior of immature ticks [105, 110]. Immature *I. scapularis* ticks in the south were most abundant on lizards, particularly skinks, which are poor *Bo. burgdorferi* reservoirs and were scarce on white-footed mice and other rodents [110, 116]. Consequently, nymphal *I. scapularis* had very low *Bo. burgdorferi* infection rates. Additionally, nymphal *I. scapularis* quest on top of the leaf litter in the north, while in the south, the nymphs are found almost exclusively inside the leaf litter or under the leaves [117]. This difference in questing behavior also contributes to lower human exposure to immature blacklegged ticks in the south. There might be genetic differences between southern and northern populations of *I. scapularis* [118].

##### Role of white-tailed deer

Many early researchers emphasize the central role of WTD in the emergence of tick-borne pathogens transmitted by *I. scapularis* [22, 107–109, 119, 120]. The increase in Lyme disease and other infections transmitted by blacklegged ticks has paralleled the explosive growth of WTD populations [108, 109, 121]. This was confirmed by early studies demonstrating that adult blacklegged ticks were found mostly on WTD [109, 114, 122]. Questing blacklegged tick populations were almost invariably associated with WTD, whereas sites without WTD had no to very low questing or attached immature tick densities [123–125]. Drastic reductions or eliminations of WTD on islands were accompanied by gradual but significant declines in tick populations [126, 127]. However, it was also demonstrated that WTD did not serve as a *Bo. burgdorferi* reservoir in an endemic area, and the ticks feeding on WTD did not acquire the pathogen [128]. White-tailed deer sera were highly lytic to *Bo. burgdorferi*, suggesting complement-mediated killing of the spirochete [129]. These findings led to the designation of WTD as an essential amplification host [114], a keystone host [22], or a reproductive stage host [130, 131]. Consequently, high densities of WTD supported blacklegged tick’s high population levels, dispersal, and intensified spread in recent decades [119, 120], maintaining *Bo. burgdorferi* enzootic transmission [22, 105, 126] and increasing the risk of Lyme disease in humans [125, 132]. An alternative viewpoint had also emerged that attributed the increase in extent and intensity of Lyme disease primarily to changes in small mammal populations rather than to those of WTD [133–135]. Lyme disease risk was correlated with biotic factors regulating rodent populations, such as the density of small-mammal predators such as coyotes and foxes [135] or acorn production [134]. In large part, these assertions were driven by the dearth of studies demonstrating a direct relationship between WTD abundance and Lyme disease epidemiological and entomological risk, particularly linking WTD densities with human exposure and disease incidence [55, 131]. Such evidence, however, has been furnished by more recent studies. Surveying islands in southeastern Canada with variable WTD and small mammal populations, a strong positive association was found between immature tick abundance, *Bo. burgdorferi*-infected nymphal tick density, and WTD abundance [136]. A more direct association with human Lyme disease was found in the Midwestern US, where Lyme incidence was closely spatially associated with WTD density and the infected ticks [137]. Perhaps the strongest evidence came from a long-term study on the effects of WTD herd culls in Connecticut [53]. Reducing the initial WTD density by nearly 90% to 5.1 WTD per square kilometer resulted in a 76% reduction in tick abundance, a 70% reduction in the entomological risk index, and an 80% reduction in resident-reported cases of Lyme disease in the community. Based on this study and other data from partial WTD culls [46, 138–140], it was suggested that maintaining densities < 3–5/km^2^ would lead to a significant reduction in the incidence of Lyme disease [54, 55].

The current scientific consensus is that only WTD can support significant *Ixodes* tick populations [130]. At high WTD densities, tick populations may be more dependent on the host species for immature tick stages, such as rodents. The precise relationship among WTD density, tick density, and Lyme disease risk has yet to be fully elucidated.

### Other tick-borne pathogens with similar transmission cycle

White-tailed deer serve as a reproductive host for two key native vector tick species, *I. scapularis* and *A. americanum*, as well as for the introduced *H. longicornis* [20, 65, 66, 136–138, 141–143]. A growing number of human pathogens transmitted by *I. scapularis* in a cycle similar to that of *Bo. burgdorferi* have been identified in the last 4 decades [144, 145]. Often, multiple pathogens co-infect the same individual ticks; therefore, it is reasonable to assume that WTD performs a similar role as an amplifier in the tick-rodent pathogen cycle [144]. Two of these pathogens are particularly widespread. Human granulocytic anaplasmosis (HGA), formerly known as human granulocytic ehrlichiosis, was first isolated in 1990 from a fatal case in the Midwestern US [146]. Human granulocytic anaplasmosis is caused by *Anaplasma phagocytophilum*, the most prevalent pathogen within Anaplasmataceae [20, 147, 148]. Since the disease became reportable in 2000, the number of cases has increased nearly 25-fold, from 273 cases in 2000 to a peak of 6729 in 2021, averaging about 2155 reported cases annually in 21 years, and the actual number of cases might be much higher [148]. In an endemic area, about 26% of adults had evidence of HGA, with the highest observed annual incidence rate of 51 per 100,000 [149]. *Anaplasma phagocytophilum* circulates in nature between blacklegged ticks and white-footed mice [150]. Short-tailed shrews and eastern chipmunks have also been reported as competent reservoirs of the pathogen, while skunks and opossums might play a minor role in the pathogen’s maintenance [151]. Although WTD are not reservoirs for strains that cause human disease, these cervids can harbor other *A. phagocytophilum* strains [152].

Human babesiosis, caused by intraerythrocytic *Babesia microti* species, was the first human pathogen attributed to *I. scapularis* ticks [104, 114] and remains one of the most common tick-borne diseases in the northeastern US [153]. Over the last decade, slightly over 2000 human cases have been reported to the CDC annually, with an incidence rate of 0.8–1.0/100,000 [154]. The incidence rate in risk groups such as the elderly can be much higher (5.0/100,000), and the true disease prevalence remains underreported [153]. The transmission cycle of *Ba. microti* is very similar to that of *Bo. burgdorferi*, the causative agent of Lyme disease [104], and co-infections in the same tick are frequently detected [144]. Likewise, WTD serves as the amplifying host for the pathogen [114, 123].

Lone star ticks transmit a number of pathogens, for which WTD serves as a reproductive host, maintaining high pathogen densities. Most notable among those are two viruses, Heartland and Bourbon. Heartland virus (*Bandavirus heartlandense*, Phenuiviridae) was isolated in 2009 from two patients, with more confirmed cases detected over the following years [155]. Heartland virus is a phlebovirus initially detected in field-collected host-seeking nymphal and adult lone star ticks in the Midwestern US [156, 157] but with human cases reported from other areas such as Virginia [158]. Horizontal, vertical, and transovarial transmission of the Heartland virus by lone star ticks was observed under laboratory conditions; however, despite several studies, no natural reservoir for the virus has been identified [157, 159]. White-tailed deer failed to develop an acute infection after inoculation with Heartland virus, leading to the conclusion that these cervids are not a likely reservoir for this virus [160].

Bourbon virus, or *Thogotovirus bourbonense*, was first isolated and recognized from a Kansas resident with severe febrile illness with a history of tick bite in 2014 [161]. To date, clinical cases are rare [162]. Bourbon virus was detected in *A. americanum* on multiple occasions and thus is the implicated vector [162–164]. Experimental infections of the putative vector, *A. americanum*, demonstrated transstadial, cofeeding, and vertical transmission of the virus [165]. The role of WTD in Bourbon virus transmission is unclear, although these cervids had a high serological prevalence of antibodies [162, 166]. In addition, the Bourbon virus was isolated from the invasive *H. longicornis* [166]. WTD is likely to serve as a reproductive host for *H. longicornis* ticks, comparable to this tick’s native host, sika deer (*Cervus nippon* Temminck) in Japan [167]. In the US, WTD can support very high densities of *H. longicornis*, suggesting a similar role [168]. WTD involvement with *H. longicornis* is discussed in more detail in the following section on WTD as a dispersal rather than the reproductive host.

### White-tailed deer as a dispersal host

#### Representative tick-borne human disease: *Rickettsia parkeri* rickettsiosis

##### Public health impacts

*Rickettsia parkeri* is transmitted by a bite of the Gulf Coast tick (*Amblyomma maculatum*) [47, 169, 170]. Between the first confirmed human infection with *R. parkeri* in 2004 and 2016, a total of 39 human cases had been confirmed [171]. However, the true incidence of *R. parkeri* rickettsiosis, also known as “American Boutonneuse fever” in the US [172], remains unknown and likely underreported because of similarities to other rickettsioses, potentially reaching thousands of human cases [47, 171]. Both the vector and the pathogen have been rapidly expanding into new geographic areas [173–178] where the disease is yet to be fully recognized.

##### Pathogen

*Rickettsia parkeri* was confirmed as a human pathogen relatively recently, although the organism was isolated in the 1930s [47, 169]. It is closely related to Spotted Fever Group rickettsiae, including the causative agent of Rocky Mountain spotted fever. Because antigenic cross-reactivity exists among rickettsiae, most serological assays are not capable of distinguishing *R. parkeri* from other *Rickettsia* species [169, 171]. However, the presence of eschar at the site of the tick bite represents a prominent clinical sign for *R. parkeri* infections.

##### Vector

The Gulf Coast tick is a three-host species distributed throughout the Americas, including the southern and mid-Atlantic US [47, 170, 179]. Recently, *A. maculatum* carrying *R. parkeri* has been reported to be expanding its range into the northeastern US [173–176, 178] and upper Midwestern US [177]. In the US, the Gulf Coast tick has very broad host preferences, having been collected from at least 71 species of birds and mammals [170]. Immature stages feed mostly on birds and small mammals, while adult ticks prefer large-bodied domestic and wild mammals [47, 170].

##### Transmission cycle

The natural history of *R. parkeri*, including the enzootic transmission cycle and reservoir species, has not been completely understood [47]. Rodents, specifically those from the two subfamilies Sigmodontinae (hispid cotton rats, *Sigmodon hispidus*, and marsh rice rats, *Oryzomys palustris*) and Arvicolinae (meadow voles, *Microtus* spp.), were proposed as potential reservoir species [180]. These rodent species hosted most immature *A. maculatum* [48, 180] and were also infected with *R. parkeri* [180]. In the laboratory study, one-third of the calves exposed to *R. parkeri* by injection or by feeding *R. parkeri*-infected *A. maculatum* were transiently rickettsemic [181]. The prevalence of *R. parkeri* in Gulf Coast ticks usually ranges from ~ 20 to 40% but can reach up to 55–60% in some locations [180, 182, 183].

##### Role of white-tailed deer

The Gulf Coast tick does not have a close association with WTD compared to the lone star or the blacklegged ticks [170]. Apart from WTD, *A. maculatum's* main host species include feral hogs, cattle, and dogs [48, 184–187]. However, *A. maculatum* is frequently collected from WTD across its range, and the infestations with this tick species can range from 20 to 100% [47, 48]. In some areas, such as northern Florida, *A. maculatum* prevalence on WTD was comparable to that of *I. scapularis* and *A. americanum* [184]. There is some evidence that *A. maculatum* infestations on the WTD have been increasing over the last decades [47, 185]. White-tailed deer are among the most important species for *A. maculatum* dispersal and establishment in new areas [48], and thus these cervids appear to act as a dispersal host, sustaining *A. maculatum* populations and facilitating the *R. parkeri* transmission cycle.

### Other tick-borne pathogens with similar transmission cycles

An exotic tick species new to the US, *H. longicornis*, was first detected in Hunterdon County, New Jersey, in 2017 [188]. Recent studies identified several species of medium- to large-sized animals as the main hosts for all stages of *H. longicornis* in North America [141–143, 168]. In urbanized New York City, raccoons, opossums, and WTD serve as hosts for *H. longicornis* [142, 143]. Unlike smaller mammalian hosts, WTD can support very high densities of *H. longicornis*, with > 1000 ticks per animal, much higher than any alternative hosts, thus serving as the main dispersal and likely reproductive host for this tick species [168]. The contribution of WTD to *H. longicornis* dispersal might be particularly significant since this tick species has rarely been found on birds [142, 168]. Multiple tick-borne pathogens of human health concerns have been detected in *H. longicornis*, although the role of this tick species in their transmission remains uncertain [141, 166, 189, 190]. Under laboratory conditions, *H. longicornis* vectored *Rickettsia rickettsii* [191] and were able to acquire POWV when cofeeding with *I. scapularis* [87]. Given the strong association of *H. longicornis* with WTD, it is plausible that WTD may also serve as a reproductive and/or reservoir host for some of the pathogens acquired by this tick species during invasion and expansion into the North American ecosystem.

### Conclusions: tick-borne pathogens and WTD

White-tailed deer play a critical role as both reservoir and reproductive hosts for various tick species, significantly influencing the transmission dynamics of numerous tick-borne pathogens (summarized in Table 1). Their ability to support high densities of ticks enhances the risk of human exposure to these pathogens, as evidenced by the correlation between WTD populations and increased incidence rates of Lyme disease [53, 192] or a rapid increase of human diseases transmitted by *A. americanum* [20]. Moreover, WTD serve as important dispersal hosts, facilitating the expansion of tick populations, including emerging species like *H. longicornis*, which may further complicate public health strategies. Understanding the multifaceted roles of WTD in these ecological interactions is essential for effective surveillance and management of tick-borne diseases [54].

**Table 1:** Pathogens and tick vectors for which white-tailed deer serve as reservoir, reproductive, or dispersal host species

Pathogen	Vector(s) that feed on WTD	WTD role	References
*Ehrlichia chaffeensis, E. ewingii*	*Amblyomma americanum*	Reservoir	[20, 56–58, 64–66, 75, 76]
*Borrelia miyamotoi*	*Ixodes scapularis*	Reservoir?/reproductive	[23, 77, 78]
Powassan virus	*Ixodes scapularis*	Reservoir?/reproductive	[23, 82–84, 86]
*Borrelia burgdorferi*	*Ixodes scapularis*	Reproductive	[88–90, 103, 107–111, 121–125]
*Anaplasma phagocytophilum*	*Ixodes scapularis*	Reproductive	[146–148, 152]
*Babesia microti*	*Ixodes scapularis*	Reproductive	[104, 114, 123, 153]
Heartland and Bourbon viruses	*Amblyomma americanum*	Reproductive	[155–166]
Multiple?	*Haemaphysalis longicornis*	Reservoir?/reproductive?	[141–143, 166, 168, 189, 190]
*Rickettsia parkeri*	*Amblyomma maculatum*	Dispersal	[47, 48, 170, 173–176, 178, 185]
Multiple?	*Haemaphysalis longicornis*	Dispersal	[141–143, 166, 168, 189, 190]

Question marks highlight research areas requiring additional study

## White-tailed deer relationship with mosquitoes and mosquito-borne pathogens

### White-tailed deer as reservoir hosts

#### Representative mosquito-borne human disease: Jamestown Canyon encephalitis

##### Public health impact

Jamestown Canyon virus (JCV) is in the *Orthobunyavirus* genus and serologically falls within the California serogroup [193, 194]. While JCV had a relatively low annual incidence of approximately 0.01 cases per 100,000 human population in 2019 and 2020 [195, 196], it is the only virus in the California serogroup that utilizes WTD as amplifying hosts. Jamestown Canyon virus was first isolated from a pool of *Culiseta melanura* in 1961 in Jamestown Canyon, Colorado [194]. In 1980, JCV was first causally linked to central nervous system disease in the case of an 8-year-old girl from Michigan [197]. Since then, serological evidence of JCV exposures in humans has been found in multiple states in the US, including New York, Connecticut, Michigan, Montana, and Alaska [197–202]. These seroprevalence rates highlight the likelihood of most JCV exposure resulting in asymptomatic or subclinical disease. As described previously, symptomatic infection may present as a non-specific febrile illness or a neuroinvasive syndrome characterized by meningitis or encephalitis. Though documentation of mortality from JCV is uncommon, long-term neurological sequelae have been reported [203–205]. Like other encephalitic arboviruses, the only available treatment for severe Jamestown Canyon disease is supportive care.

##### Pathogen

Jamestown Canyon virus (*Orthobunyavirus jamestownense*), like other *Orthobunyavirids* within the California serogroup, has a virion with three negative-sense RNA segments. Genome reassortment in nature has been documented for a growing number of viruses in the family *Peribunyaviridae*, and recent analysis of viruses in the California serogroup indicates that while segment reassortment has potentially occurred in the serogroup, it is unlikely to be a driving force in generating genomic diversity [206–210]. Previous analysis of 40 years of JCV isolates from Connecticut identified three distinct clades, A, B1, and B2. Viruses from both lineages were determined to co-circulate geographically, which further potentiates the possibility for heterologous reassortment [211]. Lineages A and B have been detected in New York, with lineage A being predominant [212]. Lineage A was also recognized in an individual JCV isolate from Maine [213].

##### Vector and transmission

Jamestown Canyon virus persists in an enzootic cycle between mosquitoes and WTD [214–216]. Similarly, the implicated breadth of potential vectors is sizeable. A number of snowmelt *Aedes* species, which overwinter as eggs and start to hatch and mature as larvae in melting snow in mid-April and hatch as adults in mid-May, are implicated in JCV cycles in spring and early summer. These mosquitoes tend to be univoltine, or hatching one brood per season, and therefore tend to have population peaks in late spring and taper off through July [217, 218]. Like other orthobunyavirids, it is likely that transovarial transmission plays an important role in the eco-epidemiology of JCV and contributes to early season transmission by snowmelt mosquitoes [219]. Evidence of vertical transmission has been detected in *Aedes provocans* and *Aedes stimulans* in addition to other early season *Aedes* spp. [220–224]. Virus overwintering in eggs likely facilitates the initiation of seasonal transmission and horizontal transmission to WTD in the spring. Early season univoltine mosquitoes frequently implicated in the transmission of JCV include *Ae. canadensis*, *Ae. provocans*, *Aedes aurifer*, *Aedes* *abserratus*, *Ae. stimulans*, *Aedes punctor*, *Aedes sticticus*, *Aedes cinereus*, and *Aedes excrucians,* among others [220, 221, 223, 225–228]. Because of the challenges in the morphological identification of these similar species, recent studies have highlighted the utility of DNA barcoding methods for more precise identification [226].

Seasonal testing in Connecticut and New Hampshire indicates that as these early snowmelt populations decline, the abundance of potential vectors such as *Cq. perturbans* and *An. punctipennis* increases [226, 228]. However, it is important to note that the relative contribution of vectors and seasonality can vary by location. Similarly, vector competence has been demonstrated for a number of univoltine snowmelt mosquitoes, including *Aedes cataphylla*, *Ae. hexodontus*, *Ae. tahoensis*, *Ae. provocans, Ae. stimulans*, *Ae. clivis*, and *Ae. increpitus* [221, 229, 230]. Studies in New Hampshire found that 95% of blood meals from putative mosquito vectors were derived from mammalian hosts, while 55% of blood meals in putative vectors, including *Ae. abserratus/punctor*, *Ae. canadensis*, *Ae. excrucians*/*stimulans*, *Ae. sticticus*, *An*. *punctipennis*, and *Cq. perturbans*, were obtained from WTD [226]. Other studies have also demonstrated the role of WTD as a blood meal source for putative JCV vectors [51, 221, 231]. *Anopheles punctipennis*, *Ae. excrucians*/*stimulans*, and *Cq. perturbans* fed on humans and could potentially serve as epizootic vectors [226].

##### Role of white-tailed deer

Blood meal analysis has demonstrated that frequently infected JCV vectors utilize WTD as a source of sustenance, as described above. Jamestown Canyon seroprevalence has been demonstrated in multiple cervid species, including WTD, sika deer, black-tailed deer, mule deer, elk, and moose, in addition to cottontail rabbits and horses [214, 232–234]. Seroprevalence rates in WTD vary significantly but have been reported as high as 91% in California [235]. Multiple studies have demonstrated an age-dependent seroprevalence trend where younger WTD have demonstrably lower rates than older individuals, making it difficult to estimate the overall prevalence of JCV in WTD [235–238]. Experimental infections in WTD demonstrated JCV viremia, indicating that these animals serve as reservoir hosts for the virus [215, 216]. An isolate of JCV from an infected WTD in Wisconsin during 1971 further reinforces the role of WTD in the enzootic maintenance of the virus [239].

#### Representative mosquito-borne human disease: cache valley encephalitis

##### Public health impact

Cache Valley virus (CVV), or *Cache Valley orthobunyavirus,* was originally isolated in 1956 from a pool of *Culiseta inornata* mosquitoes identified in Utah [240]. Cache Valley virus was first recognized as a pathogen of livestock disease during an outbreak in sheep in 1987 and later as a source of human illness in 1997 [241, 242]. Historically, CVV has not been a significant source of human disease, with only six documented cases [242–247] and one incidence of transfusion-transmitted infection [248]. Of the reported naturally acquired cases, three occurred in immunocompetent individuals, while the remaining three affected individuals with known immunocompromising conditions or pre-existing conditions that may have impacted immunoreactivity. While mild illness can include myalgia, fever, chills, headache, rash, vomiting, and fatigue, among other symptoms, severe disease typically manifests with neurological symptoms such as meningitis or encephalitis [242–248]. Serological methods on cerebrospinal fluid (CSF), serum, or tissue specimens are the primary tools for diagnosis given the transient or inapparent viremia in immunocompetent individuals. Historically, the primary assay for Cache Valley disease has been the plaque reduction neutralization assay (PRNT); however, an IgM antibody capture enzyme-linked immunosorbent assay (MAC-ELISA) is being developed [249, 250]. Currently, there is no prescribed clinical treatment or available CVV vaccine for humans.

##### Pathogen

Cache Valley virus is classified in the Bunyamwera serogroup and has the characteristic viral features of other viruses in the *Orthobunyavirus* genus, including an enveloped virion and tripartite genome as described in previous sections [250, 251]. Cache Valley virus strains delineate into two distinct lineages, though evidence of recombination between strains of both lineages has been documented [252, 253]. Evidence of CVV circulation has been observed in Mexico, four Canadian provinces, and at least 22 US states [253–260].

##### Vector

While CVV has been frequently isolated from *An. punctipennis*, *An. quadrimaculatus*, *Aedes sollicitans*, *Cq. perturbans*, *Cs. inornata*, *Aedes trivitattus*, and *Ae. canadensis*, only *Ae. sollicitans*, *An. punctipennis*, *An. quadrimaculatus*, *Cq. perturbans*, and *Cs. inornata* have been shown to be competent vectors in the laboratory [253, 261–264]. Given the large number and range of associated mosquitoes, it is difficult to implicate any primary contributors; however, one general characteristic of these potential vectors is that they all utilize mammals to some extent [51, 52, 265–267]. Specifically, in areas of New York, New Jersey, and New Hampshire, *An. quadrimaculatus*, *An. punctipennis*, *Ae. sollicitans*, and *Cq. perturbans* have been shown to take anywhere from 51 to 97% of their blood meals from WTD [51, 52, 226].

##### Role of white-tailed deer

Similar to JCV, WTD have been primarily implicated as the reservoir hosts for CVV [235, 268, 269]. Serological studies of WTD have shown a seropositivity rate ranging from 16 to 79% and have demonstrated the same age-dependent trend as seen with JCV virus in that younger animals that have had less opportunity to be exposed have lower rates [235, 268–270]. Experimental inoculation yielded a viremia of 3 log_10_ PFU/ml that was detected between days 1 and 3 post-infection [268]. This suggests that a competent mosquito could acquire the virus from an infected WTD; however, no experimental work demonstrating vector acquisition of the virus from WTD has been performed.

### White-tailed deer as reproductive host

#### Representative mosquito-borne human disease: Eastern equine encephalitis

##### Public health impacts

Eastern equine encephalitis virus (EEEV; *Togaviridae, Alphavirus*) is a highly pathogenic zoonosis transmitted by mosquitoes that causes severe disease outbreaks in humans and equines intermittently in the eastern US when ecological and environmental conditions support virus amplification. In recent years, changes in the frequencies of disease outbreaks have been recorded with a recurring annual emergence. The first human disease cases of EEE were identified by an outbreak in 1938 in southern Massachusetts [271, 272], and later disease outbreaks have occurred in at least 29 states primarily located along the East and Gulf Coasts as well as states in the upper Midwest and Canada [273]. Though humans are considered dead-end hosts as they are unable to generate a viremia high enough for acquisition by a naïve mosquito vector, humans are susceptible to developing severe neurological disease. Early systemic illness can consist of acute fever, chills, malaise, myalgia, and arthralgia, while severe neuroinvasive disease can also include headaches, altered mental status, seizures, drowsiness, and coma [274]. While an estimated < 5% of infected individuals progress to severe neurological disease, the estimated case fatality rate is 30%, though it varies from outbreak to outbreak [272, 274, 275]. Additionally, neurological sequelae afflict approximately 50% of survivors, and the estimated expenses for managing a single patient’s long-term neurological sequelae have been projected at 3 million dollars [276, 277].

##### Pathogen

Eastern equine encephalitis virus is a member of the *Alphavirus* genus in the *Togaviridae* family that was first isolated and recognized to be distinct from Western equine encephalitis virus (WEEV) in 1933 [278, 279]. Historically, EEEV was divided into four phylogenetic lineages. However, lineages II, III, and IV, which were primarily responsible for illness in horses in Central and South America, were determined to be genetically distinct and reclassified in 2010 as Madariaga virus (MADV) [280]. Alphaviruses have a single-stranded positive-sense ~ 11-kb genome with four nonstructural proteins encoded by an open reading frame in the 5’ region of the genome. The structural polyprotein is translated from a subgenomic (26S) RNA and cleaved into three major structural proteins [281].

##### Vectors and transmission

The virus perpetuates in an enzootic cycle involving mainly the black-tailed mosquito, *Cs. melanura*, and wild Passeriformes birds in freshwater hardwood swamps. Human and equine epizootics of EEEV might be facilitated by *Cs. melanura*, albeit at a low frequency, while mosquito species that bite both avian and mammalian species and are considered “bridge” vectors are believed to facilitate the emergence of the virus from the enzootic cycle. These mosquito species may include *Cq. perturbans*, *Ae. sollicitans, Ae. canadensis, Culex erraticus*, and *Uranotaenia sapphirina*, among others [282]. Vector-host interaction studies throughout the eastern US indicate that these bridge vector mosquitoes feed on several avian, mammalian, and reptilian species, with WTD as the most frequently (80%–95%) identified mammalian hosts [49, 51, 283]. In addition, blood feeding of *Cs. melanura* on WTD at lower frequency has also been reported from Connecticut (6.3%) [49], New York (3.5%) [50] Massachusetts (0.6%,) [284], Virginia (0.5%) [285], and Vermont (0.3%) [286]. However, the role of WTD in transmission dynamics is not fully explored, these investigations indicate frequent exposure by serving as a source of blood meal for EEEV-competent and infected mosquito vectors.

##### Role of white-tailed deer

The contribution of WTD to the ecology and transmission dynamics of EEEV is not well characterized, though serological evidence of WTD exposure to EEEV has been reported from 11 states and Canada [287–297]. While evidence suggests EEEV infection in WTD to be largely subclinical, there have been instances where fatal virus infections have been reported in these animals. Clinical disease was first documented in a single animal in Georgia in 2001 and subsequently in multiple free-ranging WTD in Michigan in 2005 [295, 298]. Though pathological examination indicated signs of encephalitis and meningitis in all eight total afflicted animals, the lack of other reports of EEE disease in WTD and the consistent seroprevalence levels found in WTDs in multiple geographic locations suggest that while WTD are frequently exposed to EEEV, the occurrence of clinical disease is rare [295, 298, 299]. White-tailed deer serve as a preferred blood meal source for many potential bridge vectors of EEEV. Examples include *Ae. abserratus*, *Ae. canadensis*, *Ae. cinereus*, *Ae. stimulans*, *Aedes thibaulti*, *Cx. erraticus*, *An. punctipennis*, *An. quadrimaculatus*, and *Cq. perturbans* [283, 300]. *Coquillettidia* *perturbans* has also been found to frequently feed on birds such as the American robin, black-capped chickadee, and tufted titmouse, which makes it a fitting bridge vector [283, 300]. These findings suggest that while WTD probably do not contribute to the EEEV ecological cycle, they are exposed to the virus through the bite of infected mosquitoes acquiring a blood meal.

#### Representative mosquito-borne human disease: West Nile

##### Public health impact

West Nile virus (WNV; *Flaviviridae*, *Orthovirus*), a virus with global distribution, is the primary cause of mosquito-borne disease in the continental US, with 59,141 reported human disease cases between 1999 and 2023 [301]. West Nile virus was first isolated from a female patient in Uganda in 1937 [302], and in 1999, the virus emerged in New York, USA, and rapidly spread across the country and into neighboring countries as well as the Caribbean and Central America [303, 304]. Lineage I strains of WNV are the circulating strains causing disease in North America, with the highest incidence of neuroinvasive disease occurring in individuals ≥ 70 years of age [305]. From 2009 through 2018, the peak incidence rate of WNV neuroinvasive disease in the US was 0.92 per 100,000 in 2012; however, the average over the 9 years was just 0.44 per 100,000 [305]. Similarly, in 2019, the incidence of neuroinvasive disease was only 0.19 per 100,000, which highlights the significant variability of disease incidence over time [196]. It has been estimated that up to 80% of all WNV infections are asymptomatic, resulting in a likely underestimation of case numbers [306]. During the evaluation of WNV from 2009 to2018, the case fatality rate was reported at 5%; however, due to fatality rates multiplying with increasing subject age and the unknown number of unreported subclinical cases, the overall case fatality rate is difficult to discern [305, 307, 308]. Similarly, of those who develop symptoms, most experience a self-limiting disease that may entail acute fever, headache, fatigue, malaise, muscle pain and weakness, gastrointestinal symptoms, and/or a transient macular rash [309]. Neuroinvasive disease afflicts an estimated 1% of individuals infected and manifests as meningitis, encephalitis, or paralysis. Patients who develop severe disease are also at risk for long-term sequelae, which can manifest as physical, cognitive, or functional difficulties such as extreme fatigue, movement disorders, pain, and cognitive decline [310]. While there is currently no licensed vaccine for humans, vaccines are available for horses and domestic geese [311].

##### Pathogen

West Nile virus, or *Orthoflavivirus nilense*, is classified in the family *Flaviviridae* and genus *Orthoflavivirus* [193]. The enveloped virus consists of a positive strand genome with two terminal untranslated regions on the 5’ and 3’ ends and a single open reading frame that encodes three structural proteins (capsid, premembrane, and envelope) and five nonstructural proteins [304].

##### Vectors and transmission

Like EEEV, WNV is maintained in an enzootic cycle involving multiple mosquito species, though primarily a few species in the genus *Culex* and wild Passeriformes birds. In the US, the primary vectors vary by geographic region, season, local ecology, and climate [312]. For instance, in the northeastern, mid-Atlantic, and central US, *Cx. pipiens* and *Culex restuans* (as an enzootic vector) have emerged as the primary vectors, whereas in the southwest, *Cx. quinquefasciatus* is largely responsible for urban transmission, while *Culex tarsalis* often serves as the vector in rural areas [49, 311–316]. *Culex salinarius* is also a WNV vector recognized along the east coast that feeds on both avian and mammalian hosts, including humans, a characteristic that qualifies this mosquito species as a bridge vector that utilizes mammals, including humans, as hosts at high population densities [50, 312, 317]. Another bridge vector primarily active in the southeastern and Gulf Coast regions, *Culex nigripalpus*, utilizes freshly flooded ditches for immature habitat. *Culex nigripalpus* shifts from feeding largely on birds in the early season to becoming more of an opportunist in the later season, which supports its role as a bridge vector [317–319]. Vector competence studies in California also highlighted *Cx. stigmatosoma* and *Cx. erythrothorax* as efficient laboratory vectors, though each is believed to play different roles in the ecological cycle [320–324]. In addition, several other competent mosquito species, such as *Ae. vexans*, *Aedes albopictus*, *Aedes japonicus*, and *Aedes trivittatus*, have also tested positive for WNV and are likely involved in WNV transmission as bridge vectors [312].

##### Role of white-tailed deer

Given the rare case of overt disease and the extensive WNV seroprevalence documented, WTD are generally considered dead-end hosts unable to generate sufficient viremia for mosquito acquisition [325]. As such, WTD serves as a source of sustenance for primarily mammalophilic bridge vectors of WNV such as *Ae*. *vexans* and *Cx. salinarius* [49, 50, 317]. Only one fatal case of WNV was reported in a WTD in Georgia in 2005 [326]. To date, there have been no studies examining the experimental infection of WTD; however, there is an abundance of data demonstrating the prevalence of neutralizing antibodies to WNV. A serosurvey study from 1999 to 2003 was able to capture the introduction of WNV into Iowa following a seropositivity increase > 23.3% in 2002 following the 2001 introduction to the state [327]. Similarly, the use of WTD as sentinels for WNV in New Jersey identified the presence of a circulating virus in 2001 [328]. The information gleaned from these surveys in the years leading up to and immediately following the introduction of WNV to the US reinforces previous assertions touting the potential utility of using wildlife as sentinels [291, 329]. Information regarding the geographic range of WNV throughout the US in the years since has been well supplemented using additional serosurveillance studies [84, 290, 330].

#### Representative mosquito-borne human disease: La Crosse encephalitis

##### Public health impact

La Crosse virus (LACV), *Orthobunyavirus lacrossense*, falls within the *Orthobunyavirus* genus of the family *Peribunyaviridae* and is categorized into the California serogroup [331]. Members of the *Orthobunyavirus* genus have a negative-sense, single-stranded, tripartite RNA genome composed of three segments. The small segment (S) encodes the nucleocapsid [332], the medium segment (M) encodes two structural glycoproteins and a nonstructural protein designated NSm [333], and the large segment (L) encodes the RNA-dependent RNA polymerase [334]. Like other arboviruses discussed herein, *Orthobunyavirids* can cause a range of diseases, spanning from asymptomatic to mortal neuroinvasive disease. Non-neuroinvasive disease is often characterized by general signs and symptoms that could entail febrile illness, headache, myalgia, arthralgia, rash, or gastrointestinal symptoms. Neuroinvasive disease is more severe and consists of meningitis, encephalitis, acute flaccid paralysis, or other indicators of neurological dysfunction [335]. Though these viruses are difficult to distinguish serologically, their ecological cycles, geographic range, and public health impact vary considerably. La Crosse virus is the most common source of pediatric neuroinvasive arboviral disease in the US [336]. La Crosse virus was initially isolated in 1960 from a child with meningoencephalitis [337]. Historically, LACV has been responsible for the second highest number of domestic arboviral disease cases behind WNV. In 2020, LACV was responsible for 21 cases, or 2% of all neuroinvasive cases, while in 2019, this virus was confirmed in 55 (5%) neuroinvasive cases [195, 196]. Though the national average incidence of neuroinvasive LACV during the 17-year period from 2003 to 2019 was 0.02 cases per 100,000 persons, it is important to note the highly focal nature of the virus circulation. A cluster analysis looking at LACV neuroinvasive disease occurring from 2003 to2021 identified that five states were responsible for 87% of the cases: Ohio, North Carolina, West Virginia, Tennessee, and Wisconsin. Within those states, three risk cluster areas were identified [338]. The focal nature of LACV is likely linked to the ecological cycle in addition to socioeconomic predictors [338]. Three distinct phylogenetic lineages have been identified for LACV [339, 340], though analysis indicates that lineage I genotypes are associated with more severe clinical outcomes [341–343].

##### Vectors and transmission

La Crosse virus is maintained horizontally between *Aedes triseriatus*, the eastern tree-hole mosquito, and small rodents such as the eastern chipmunk [344–348]. However, an efficient transovarial cycle allowing the virus to be maintained throughout diapause and persist in the absence of horizontal transmission plays a large role in the enzootic perpetuation of the pathogen [349–351]. Venereal transmission has also been described for *Ae. triseriatus* [352]. *Aedes triseriatus* is present in hardwood forests in central and eastern US and is considered a “tree-hole mosquito,” as immature stages can be found in water-holding tree holes; however, this mosquito can also utilize containers with fresh water [353, 354]. *Aedes albopictus* and *Ae. japonicus*, which both utilize natural and artificial water containers for oviposition, are also competent vectors of LACV considered important for viral maintenance in the Appalachian region [355–360]. Host preference studies have indicated *Ae. triseriatus* largely feeds on mammalian hosts, with > 60% of the blood meals originating from WTD, though a small number have also been found feeding from avian sources [51, 265]. Additionally, humans, cats, rats, and chipmunks have been found as blood meal sources [51]. Interestingly, chipmunks were only identified as a host source for 7.6% of blood meals in a study in Wisconsin, while no chipmunks or squirrels were identified as utilized hosts in Connecticut [51, 265]. It has been well established that *Ae. albopictus* indiscriminately feeds on avian and mammalian hosts, including rabbit, rat, dog, cow, human, WTD, squirrel, raccoon, cat, and opossum, as well as reptiles and amphibians [361–365]. Alternatively, *Ae. japonicus* is a fastidious mammalian biter and has been shown to acquire a large percentage of blood meals (> 50%) from WTD; however, it has also taken blood meals from cats, dogs, rabbits, humans, cows, and horses [51, 365, 366].

##### Role of white-tailed deer

Though the primary vectors of LACV are reported to take a large proportion of blood meals from mammals, including WTD, horizontal transmission between this cervid species and *Ae. triseriatus* has never been successfully demonstrated [265, 357, 362, 364, 365]. While two studies reported that WTD acquire a short-lived low viremia following needle inoculation or exposure to infected mosquitoes, neither study demonstrated subsequent infection of susceptible *Ae. triseriatus* from an infected WTD [215, 367]. Notably, LACV transmission experiments between *Ae. japonicus* and *Ae. albopictus* and WTD have not been performed.

Surveys of LACV neutralizing antibodies in wild WTD typically demonstrate relatively low seroprevalence in comparison to that of JCV, for which WTD are recognized as the amplifying host. For instance, studies from Wisconsin, Minnesota, and California identified a seroprevalence rate ranging from 15 to 63% for JCV and < 1–16% for LACV [233, 235–238]. While a higher rate of LACV seroprevalence would be expected for an amplifying host, it would be informative to assess the seropositivity rates in areas of focal LACV circulation in regions of the Appalachians.

### Conclusions: mosquito-borne pathogens and WTD

Though WTD play important roles for the propagation of mosquitoes and potentially the viruses they transmit, the interplay between mosquitoes and these cervids as it pertains to arboviruses differs considerably from that of ticks (summarized in Table 2). For instance, unlike ticks, mosquitoes can acquire their blood meal from a host in a matter of moments, whereas ticks must persist on their host for days until they have acquired their blood meal. This highlights the irrelevance of WTD as dispersal hosts for mosquitoes compared to ticks. Similarly, many of the mosquito species considered in the above discussion have a wide range of host preferences, unlike some tick life stages, which may be highly fastidious towards WTD. Because viruses like JCV and CVV utilize WTD as amplifying hosts, many associated vectors primarily acquire blood meals from these hosts. For instance, *Ae. canadensis*, *An. punctipennis*, and *Cq. perturbans* mosquitoes have all been documented to feed on WTD at rates of > 90% [51, 227, 283, 368]. Alternatively, a number of primary and bridge vectors of EEEV, WNV, and LACV have also been documented to feed on WTD. In this case, WTD serve solely as a source of blood for reproduction as opposed to a reservoir host for virus maintenance and amplification. For EEEV, putative secondary vectors including *Ae. canadensis*, *Aedes cantator*, *An. punctipennis*, *Ae. taeniorhynchus*, and *An. quadrimaculatus* primarily utilize WTD as reproductive hosts [51, 52, 283]. However, vectors of WNV and LACV rely on WTD less frequently than the other arboviruses described herein; vectors like *Cx. salinarius* and *Ae. triseriatus* feed on WTD at moderate to high rates [50, 51, 238, 368]. Regarding arboviruses, WTD can provide a useful sentinel model allowing for surveillance of emerging viruses, as even arboviruses that do not make use of WTD as reservoir hosts will often result in seroconversion, which can be measured. This has been demonstrated with the spread of EEEV through the upper Northeast states such as New Hampshire, Vermont, and Maine, as well as WNV in Texas, among other examples [288, 294, 330, 369, 370]. Although WTD are not anticipated to play a role in the ecological cycle of BRBV or HRTV viruses, they can also be utilized to identify the emergence of these viruses into new locations. By understanding the interplay among mosquitoes, their pathogens, and WTD, we can direct surveillance programs with the ultimate goal of disease prediction and prevention.

**Table 2:** Pathogens and mosquito vectors for which white-tailed deer serve as reservoir or reproductive host species

Pathogen	Vector(s) that feed on WTD	WTD role	References
Cache Valley virus			
	*Aedes sollicitans*	Reservoir	[51]
	*Anopheles punctipennis*	Reservoir	[51, 52, 226]
	*Anopheles quadrimaculatus*	Reservoir	[51, 52]
	*Coquilletidia perturbans*	Reservoir	[51, 226, 368]
Jamestown Canyon virus			
	*Aedes abserratus/punctor*	Reservoir	[51, 226]
	*Aedes canadensis*	Reservoir	[226, 227, 368]
	*Aedes excrucians/stimulans*	Reservoir	[51, 221, 226]
	*Aedes sticticus*	Reservoir	[51, 226]
	*Anopheles punctipennis*	Reservoir	[51, 226]
	*Coquilletidia perturbans*	Reservoir	[51, 368]
Eastern equine encephalitis virus			
	*Aedes canadensis*	Reproductive	[51, 226, 283, 368]
	*Anopheles punctipennis*	Reproductive	[51, 52, 226, 283]
	*Anopheles quadrimaculatus*	Reproductive	[51, 52, 283]
	*Coquilletidia perturbans*	Reproductive	[51, 283, 368]
West Nile virus			
	*Aedes vexans*	Reproductive	[49, 368]
	*Culex salinarius*	Reproductive	[50, 368]
La Crosse virus			
	*Aedes triseriatus*	Reproductive	[51]
	*Aedes japonicus*	Reproductive	[51]
	*Aedes albopictus*	Reproductive	[362–364]

## Conclusions

White-tailed deer are long-lived mammals with a wide geographic range in the continental US whose presence has a cascading effect on environmental ecology, predator-prey dynamics, human behavior, and human and veterinary diseases. Many of these variables have downstream effects on one another. Historically, substantial changes in WTD populations, due to either habitat removal or unmitigated hunting, have supported the re-emergence of forest habitat and resulted in WTD reintroduction. Not only do WTD serve as a food source for hematophagous arthropods, but also the renewed vegetation provides a prime habitat for arthropod propagation and frequent host-seeking. It is well established that tick vectors such as the blacklegged tick (*I. scapularis)* and the lone star tick (*A. americanum)* are often associated with WTD. Notably, adult tick populations and their growing geographic range depend on WTD. White-tailed deer can serve as reproductive, reservoir, or dispersal hosts for both ticks and mosquitoes and play a vital role in enzootic and epizootic pathogen cycles. For instance, WTD deer serve as a reproductive host for *I. scapularis*, which facilitates the ecological cycle of *Bo. burgdorferi*. This is also the case for other pathogens transmitted by *I. scapularis,* such as *Ba. microti*, *A. phagocytophilum*, and POWV. Viruses such as Heartland and Bourbon viruses, transmitted by *A*. *americanum*, also depend on WTD as a propagative vector host. The reproductive role of WTD for mosquitoes involved in pathogen transmission is less often considered and is underestimated, likely because they are less fastidious and frequently use multiple hosts. For instance, while the principal vector of EEEV primarily feeds on birds, at least nine potential bridge vectors implicated in epizootic spillover utilize WTD as a preferred blood meal host. The case is similar for WNV and LACV, in that implicated vectors utilize WTD as a propagative blood source. In addition to supporting vector propagation, WTD plays an essential role in the maintenance of pathogen cycles. While WTD are known to be refractory to *Bo. burgdorferi* and have never been shown to amplify WNV, EEEV, or LACV, they act as a reservoir host for *E. chaffeensis* and arboviruses Jamestown Canyon and Cache Valley and are necessary for the enzootic persistence of the pathogens. A less recognized role of WTD is that of a dispersal host. This is exemplified by *A*. *maculatum* and a pathogen it vectors, *R. parkeri*, or American Boutonneuse fever. As this recently identified human pathogen emerges, WTD are expected to play an important role given the vector’s association with the host and their large geographic range and movement. As populations of WTD have grown and spread along with the expansion of human settlements, interactions between the two species have also increased. Additionally, the geographic movement of WTD, the introduction of new invasive arthropods that have the potential as vectors, and the emergence of novel pathogens will introduce major challenges to vector-borne disease prevention in the future. Understanding the often-overlooked role that WTD play in pathogen emergence is critical.

## Data Availability

No datasets were generated or analyzed during the current study.

## Abbreviations

US: United States
WTD: White-tailed deer
HME: Human monocytic ehrlichiosis
E.: Ehrlichia
A.: Amblyomma
Bo.: Borrelia
I.: Ixodes
CDC: Centers for Disease Control and Prevention
Ba.: Babesia
HME: Human monocytic ehrlichiosis
HGA: Human granulocytic anaplasmosis
H.: Haemaphysalis
R.: Rickettsia
POWV: Powassan virus
Cs.: Culiseta
Ae.: Aedes
Cq.: Coquillettidia
Cx.: Culex
An.: Anopheles
WNV: West Nile virus
EEE: Eastern equine encephalitis
EEEV: Eastern equine encephalitis virus
JCV: Jamestown Canyon virus
LACV: La Crosse virus
CVV: Cache Valley virus
CSF: Cerebrospinal fluid
PRNT: Plaque reduction neutralization assay
BSL: Biosafety level
MAC-ELISA: IgM antibody capture enzyme-linked immunosorbent assay
